# Precision cancer medicine in Europe: a mixed-methods study on infrastructure for extended molecular diagnostics

**DOI:** 10.1007/s00432-026-06468-y

**Published:** 2026-04-02

**Authors:** Pia S. Henkel, Kine Pedersen, Kjetil Taskén, Ebba Hallersjö Hult, Hans Gelderblom, G. Live Fagereng, Helga B. Landsverk, Eline Aas

**Affiliations:** 1 Institute of Health and Society, University of Oslo, Oslo, Norway; 2 Institute for Cancer Research, Oslo University Hospital, Oslo, Norway; 3 Institute of Clinical Medicine, University of Oslo, Oslo, Norway; 4 Stockholm School of Economics Institute for Research, Stockholm, Sweden; 5 Department of Medical Oncology, Leiden University Medical Center, Leiden, The Netherlands; 6 Research Support Unit, Oslo University Hospital, Oslo, Norway; 7 Division for Health Services, Norwegian Institute of Public Health, Oslo, Norway

**Keywords:** Precision cancer medicine, Europe, Infrastructure, Molecular diagnostics, Implementation, Next-generation sequencing

## Abstract

**Purpose:**

Precision cancer medicine (PCM), targeting cancer treatment to patients’ individual genomic profiles, has the potential to improve diagnosis and outcomes substantially. Implementing PCM in healthcare systems requires that extended molecular diagnostics are accessible as part of routine practice. We conducted a mixed-methods study to identify and inform the infrastructure necessary for the implementation of extended molecular diagnostics as part of the health care system.

**Methods:**

To identify and inform relevant categories of necessary infrastructure, we combined a survey, a structured literature search, and expert interviews. The survey included questions on reimbursement and technology assessment of diagnostics for PCM. Findings from the structured literature search were grouped to identify categories of infrastructure together with emerging themes in interviews with experienced oncologists, pathologists, and health technology assessment experts.

**Results:**

44 respondents from 20 countries participated in our survey. We identified and included 45 published papers, and seven experts were interviewed. We identified six key categories for implementing PCM as part of the healthcare system: physical, financial, organisational, competency, data and legal infrastructure. Our combined data sources revealed several themes to promote implementation, including the importance of centralisation of testing, the need for sustainable reimbursement, engagement of relevant stakeholders, interoperability of data infrastructure, and inter-disciplinary collaboration.

**Conclusion:**

Our results highlight the need for interdisciplinary approaches and the value of initiatives to establish infrastructure within and across countries. While several European countries have come a long way in implementing diagnostics for PCM, considerable work remains to ensure equitable access for patients in need.

**Supplementary Information:**

The online version contains supplementary material available at 10.1007/s00432-026-06468-y.

## Introduction

Cancer is one of the prime applications of precision medicine, where prevention and treatment of diseases are personalized to an individual’s characteristics (Collins and Varmus 2015). In precision cancer medicine (PCM), molecular analyses of a patient’s tumour can identify alterations in the tumour DNA that drive tumour growth (Schwartzberg et al. 2017). As more and more cancer treatments are designed to target these alterations, molecular diagnostics that identify actionable alterations in the tumour DNA are becoming increasingly relevant and a topic of high interest in European healthcare systems (European Commission, 2021; Malone et al. 2020). The rapid development of next-generation sequencing (NGS) technologies allowing for fast and multiple parallel analyses has further contributed to the relevance of molecular diagnostics (Malone et al. 2020; Mateo et al. 2022). The NGS panels used to sequence specific regions or genes of DNA are becoming increasingly comprehensive, up to the analysis of all the DNA in whole-genome sequencing (Edsjo et al. 2024). For several cancer patient groups, NGS is recommended as part of clinical practice guidelines reflecting the importance of molecularly guided treatment for cancer care (Mosele et al. 2024).

However, according to a 2021 survey, routine access to NGS-based diagnostics in oncology beyond single-gene tests varies across Europe (Bayle et al. 2023). Comprehensive sequencing panels were available in routine practice in a few high-income European countries, while availability in other countries did not extend beyond clinical trials and research. Reimbursement of tests and access to targeted treatments were mentioned as important barriers (Bayle et al. 2023). In another study, broader access to precision cancer medicine was associated with existing public national reimbursement processes while a lack of diagnostic facilities and inefficient organizational structures hinder access to testing (Normanno et al. 2022). Assessing patient access to molecular diagnostics is further complicated by limited reporting of actual testing rates across Europe (de Jager et al. 2024). In addition to these financial and logistical barriers, challenges to the implementation of extended molecular diagnostics include sharing and analysis of genomic data (Bertier et al. 2016), the design of precision cancer medicine trials (Bertier et al. 2016), and the value assessment frameworks for comprehensive molecular analyses guiding funding and reimbursement in many healthcare systems (Tarride et al. 2022). Finally, several studies highlight the need for multi-stakeholder collaboration when implementing precision oncology in the healthcare system (Fasola et al. 2023; Horgan et al. 2022; Mateo et al. 2022).

Based on the mentioned challenges, it appears that the infrastructure required to enable the implementation of extended molecular diagnostics can include both tangible elements, such as laboratories and transport logistics, and intangible elements, such as data systems and organisational frameworks, including reimbursement of diagnostics as part of (financial) infrastructure. We assume that with the establishment of the necessary infrastructure, extended molecular diagnostics can be implemented as part of the healthcare system, i.e. would be accessible nationwide to all eligible patients. Within the Personalised Cancer Medicine for all EU citizens (PCM4EU) project that includes 17 partners from 15 European countries (Tasken et al., 2024), we aimed to provide an overview of the infrastructure needed to integrate extended molecular diagnostics (i.e., the testing technology) into health care systems. We examined the diagnostic pathway from patient referral to reporting, considering necessary infrastructure independent of existing country-specific infrastructure in European countries.

## Methods

**Fig. 1:**
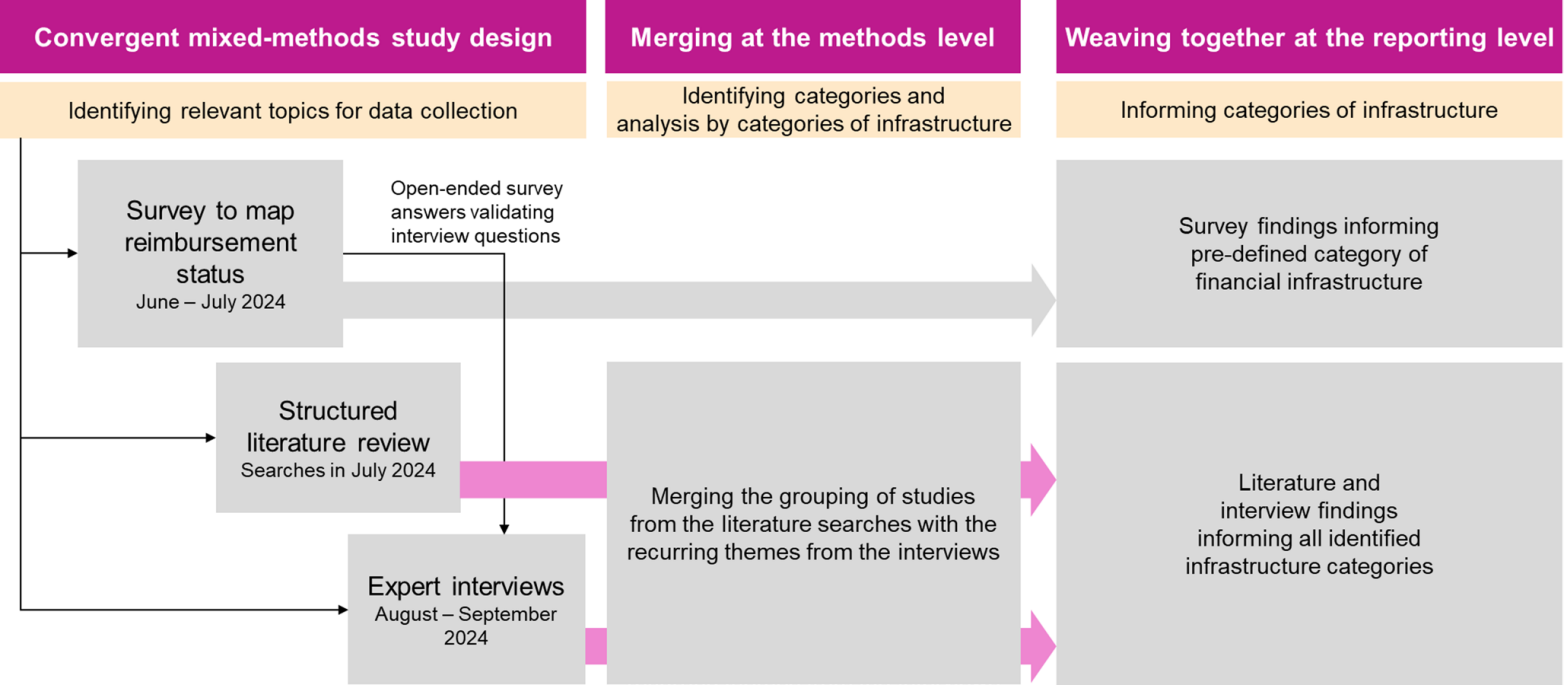
Overview of the mixed-methods research approach. This figure displays the use of a survey, structured literature review and expert interviews and their integration at the study design, methods and reporting level

Given the complex and multi-faceted concept of infrastructure within healthcare systems, we utilized a mixed methods approach that integrates different methods at the study design, methods and reporting level (Fetters et al. 2013). Therefore, we used a combined data collection approach using a survey, a structured literature search, and expert interviews, to identify categories of necessary infrastructure and inform these categories (Fig. 1).

Based on our initial literature search naming reimbursement as a key barrier, the survey included questions related to the reimbursement of five types of diagnostics: single biomarker tests, NGS targeted panels, NGS comprehensive panels, whole genome sequencing (WGS), and analysis of circulating tumour DNA (ctDNA). The survey further included questions on the health technology assessment process for diagnostics, and an open-ended question on barriers for PCM beyond reimbursement (questions in Supplemental Material 1). The survey was conducted online using SurveyMonkey and distributed via direct e-mail to PCM4EU (https://www.matrix-fkb.no/en/pcm4eu/partners, accessed November 14, 2025) and ASCERTAIN (https://www.access2meds.eu/, accessed November 14, 2025) consortium members. We encouraged the recipients to further distribute the survey and sent individual reminders to contacts from those countries for which responses were lacking. The survey was open from June 10 to July 29, 2024. We compiled responses into a consolidated reply per country and reviewed the results with members of the PCM4EU consortium with extended knowledge about the current status in their own country for validation and clarification. For two countries, the reporting of the results was altered accordingly.

To gain a broader perspective on infrastructure beyond reimbursement and financial aspects, we conducted a literature search by searching the databases Medline via PubMed and Embase via Ovid on July 16, 2024. In line with the explorative character of our study, we opted to conduct a structured literature review. We built our search query by combining the search terms *((Next Generation Sequencing OR Precision Medicine) AND Oncology OR Precision Oncology) AND Infrastructure*, as well as using related search terms and database-specific subject headings (Supplemental material 3). We limited the results to studies published in the last 10 years, given the fast-paced innovation cycles in the field of molecular diagnostics. One researcher screened titles and abstracts for relevance and subsequently assessed full-text eligibility. We excluded studies that did not specifically report on infrastructure with relevance for European countries, studies focusing on targeted treatment only, editorials and comments without research results, and conference abstracts. To avoid omitting important publications, we added relevant articles suggested by consortium members of the PCM4EU and ASCERTAIN projects that had not been identified in the structured literature search.

To complement with a broader perspective, we developed an initial list of interview questions for experts in the field of PCM, recruited through the network of the PCM4EU and the ASCERTAIN projects. We invited potential interview partners with extensive experience with molecular diagnostics in either a clinical, health technology assessment, payer or policy-related role, aiming for a diverse group of interview partners with regards to their professional role and their country. We discussed the list of questions and validated it using the responses from the open-ended survey question (Supplementary Table 2). As a result, we decided that clinical guidelines defining patient eligibility for molecular diagnostics as well as access to targeted treatment would be out of the scope of this study. Interview questions (Supplemental material 2) included the current and future use of diagnostics for PCM in the interviewee’s country or institution, necessary infrastructure, the pathway to routine care, reimbursement and funding, PCM organisation, and barriers to implementation. We conducted the semi-structured interviews between August 1 and September 26, 2024. We conducted the interviews through video meetings and subsequently used an automatic speech-to-text transcription service provided by the University of Oslo (Autotekst).

We identified our final list of infrastructure categories by merging the concepts used to group the included studies from the literature search and recurring themes in the expert interviews. The infrastructure categories were derived from the data, with one researcher iteratively analysing the transcripts of the interviews, highlighting themes and color-coding them according to categories of infrastructure. The categorization of themes into categories and the naming of the categories were subsequently discussed with two of the co-authors, and applicable changes were agreed on. Given a limited number of interviews and our explorative research scope, we did not assess thematic saturation within the identified categories. For each of these identified categories, we then weaved together the findings from the literature search and expert interviews narratively as per the mixed-methods study design (Fetters et al. 2013). The findings from the survey were additionally used to inform the pre-defined financial infrastructure category. We used the findings per category to derive comprehensive recommendations and discussed potential deviations across different methods or included countries.

This study was conducted as part of the EU-funded project Personalised Cancer Medicine for all EU Citizens (PCM4EU). The collection of data trough expert interviews was conducted in accordance with General Data Protection Regulation (GDPR) Article 6(1)(a) by obtaining written informed consent from the interviewees. In accordance with routines for processing personal data in research projects at the University of Oslo, approval for data collection was applied for and approved by the Norwegian Agency for Shared Services in Education and Research (SIKT, reference nr. 371455). The analysis of survey results required no further ethical approval as only anonymized data was handled, and survey participants were informed before participating that survey results will be published in aggregated, anonymized form. The structured literature search made use of publicly available data only. Therefore, an accordance statement is not applicable for these parts of the study.

## Results

### Sample characteristics

In total, 44 respondents from 20 countries participated in the survey (Supplementary Table 1). More than half of the respondents (*n* = 24) were oncologists. Other professions included pathologists, molecular biologists, health economists, geneticists, or coordinators (Supplementary Fig. 1). More than half of the respondents (*n* = 23) had over 5 years of experience with PCM (Supplementary Fig. 2).

Our literature search resulted in 125 records for full-text review. Eighty-three studies were excluded based on the full text review, primarily studies related to clinical implementation of precision diagnostics, reporting on clinical actionability and patient characteristics (rather than infrastructure). We included three additional expert-suggested studies about reimbursement, value assessment and a national PCM infrastructure project.

Of eleven interview requests sent out, seven experts replied, and all seven interviews were subsequently conducted. The interview partners worked at institutions in Croatia, Estonia, Finland, Germany, Lithuania, Norway, and Spain. They were working as oncologists (*n* = 3), pathologists (*n* = 2), advisors (*n* = 1), and HTA experts (*n* = 1) in university hospitals (*n* = 4), a national cancer institute (*n* = 1), a national regulatory agency (*n* = 1), or as independent specialists (*n* = 1). The interviews lasted between 45 and 65 min.

### Identified categories of necessary infrastructure

**Fig. 2:**
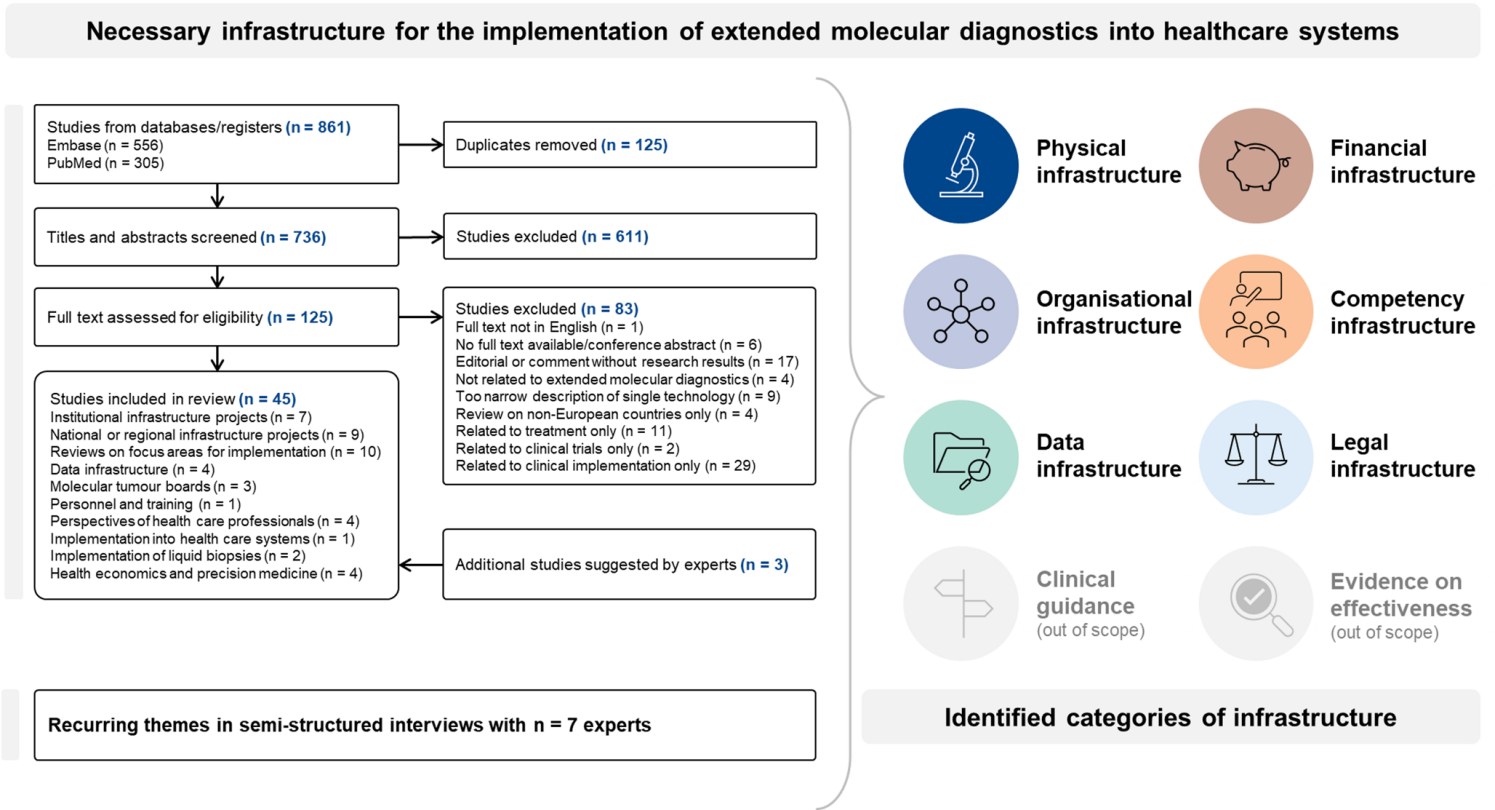
Identification of categories of infrastructure. This figure displays the flow of information through the different phases of the structured literature review on the left side. Based on the structured literature search and recurring themes in the expert interviews, categories of infrastructure were identified, which are displayed on the right side of the figure

We grouped the 45 included studies into concepts as follows: data infrastructure, molecular tumour boards or implementation into health care systems, personnel and training and the perspectives of health care professionals, implementation of liquid biopsies, and health economics and precision medicine. Furthermore, we included reviews suggesting general focus areas for implementation, infrastructure projects at one institution or a network of institutions, and national or regional infrastructure projects (thereof three related to PCM4EU). Together with recurring themes in the expert interviews (Supplementary Table 3) we then derived six categories of infrastructure: physical (including different technical applications such as liquid biopsies), financial (including reimbursement and funding), organisational (including organisation of molecular tumour boards), competency (including training and the perspectives of health care professionals), data, and legal infrastructure (Fig. 2).

#### Physical infrastructure

Healthcare institutions providing extended molecular diagnostics need to select an NGS platform. Literature suggests selection criteria, including genes covered, testing volume, DNA requirements and availability of bioinformatics systems for clinical reporting (Hynes et al. 2017). Turnaround times in clinical practice and the connection between testing volume, testing capacity, reagent shelf-life and turnaround time is also highlighted as important considerations for selecting and operating the physical laboratory equipment needed for NGS (Ijzerman et al. 2021; Koessler et al. 2019). The need for validation to prove the robustness of the diagnostic or prognostic information is emphasised, given how crucial specificity and sensitivity are for clinical utility (Koessler et al. 2019; Rolfo et al. 2020). The analysis of circulating tumour DNA has emerged as an alternative to tumour tissue. It is suggested to clarify how liquid biopsies can be integrated into the diagnostic pathway and in what situations they can replace or complement more invasive tissue-based analysis (Haselmann et al. 2022; Ijzerman et al. 2021).

In expert interviews, the contextualization of the choice of analysis technology was a recurring theme. Experts mentioned that the selection of a specific NGS platform was impacted by available resources, disease characteristics, patient preferences, access to treatment, testing volume and the need to combine samples from patients with different tumour types using the same analysis panel for institutions serving small populations. Some experts described customising gene panels to match exactly those genetic alterations for which targeted treatments are available. In contrast, others mentioned that using standardised panels would enable quick modifications to reduce costs and dependency on commercial providers of sequencing panels. The amount of tumour tissue available was mentioned as a common limitation. Performing more comprehensive molecular analysis for more patients would require changes in the biopsy surgery processes and the sample preparation. One expert summarized this contextualization as follows:

So, everything is optimized now to do the best that we can, for the patients that we have, based on the capabilities that we have.

Regarding validation, experts mentioned that lacking personnel is a challenge in validation, as non-commercial panels need to comply with the European in-vitro diagnostic device regulation.

#### Financial infrastructure

**Fig. 3:**
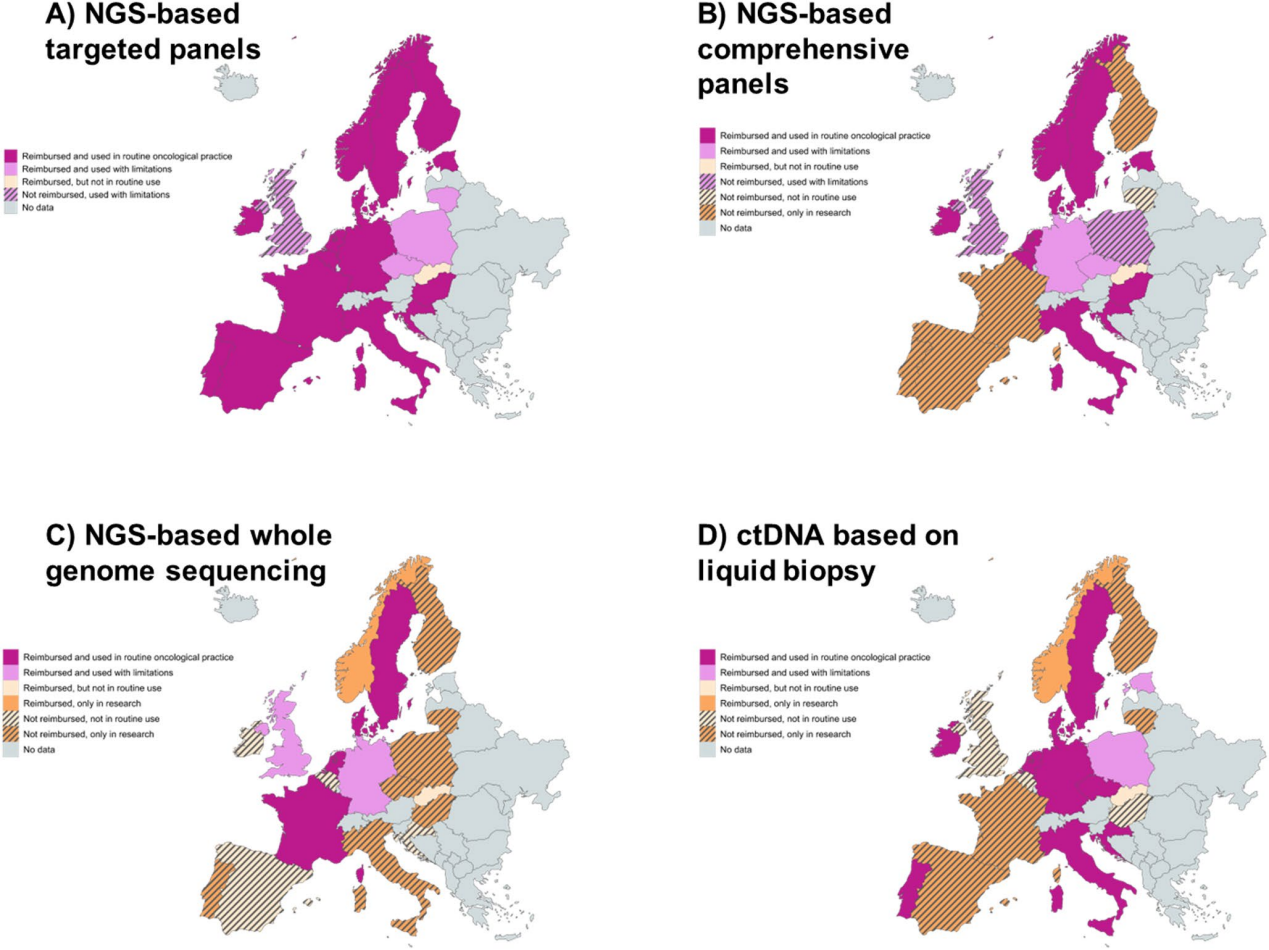
Current reimbursement status and use of diagnostics for precision cancer medicine in 20 European countries. This figure depicts the survey results on current reimbursement status and use for specific tumour types according to guidelines of **A** NGS-based targeted panels, **B** NGS-based comprehensive panels covering several hundred genes, **C** NGS-based whole genome sequencing and **D** ctDNA analysis based on liquid biopsy, reporting consolidated responses per country. In the case of multiple respondents per country, responses were consolidated, reporting a positive response, for example, “Yes, reimbursement by the national healthcare system or an insurer” if at least one respondent answered with “Yes”. “Used with limitation” refers to countries for which survey respondents specifically indicated that the test is used for specific patients only. Results reflect the situation at respondents’ institutions, rather than a standardised reimbursement decision across a whole country. For ctDNA based on liquid biopsy, no differentiation was made between the analysis of single genes or comprehensive genomic profiling based on liquid biopsy. Abbreviations: NGS = next-generation sequencing, ctDNA = circulating tumour DNA

**Fig. 4:**
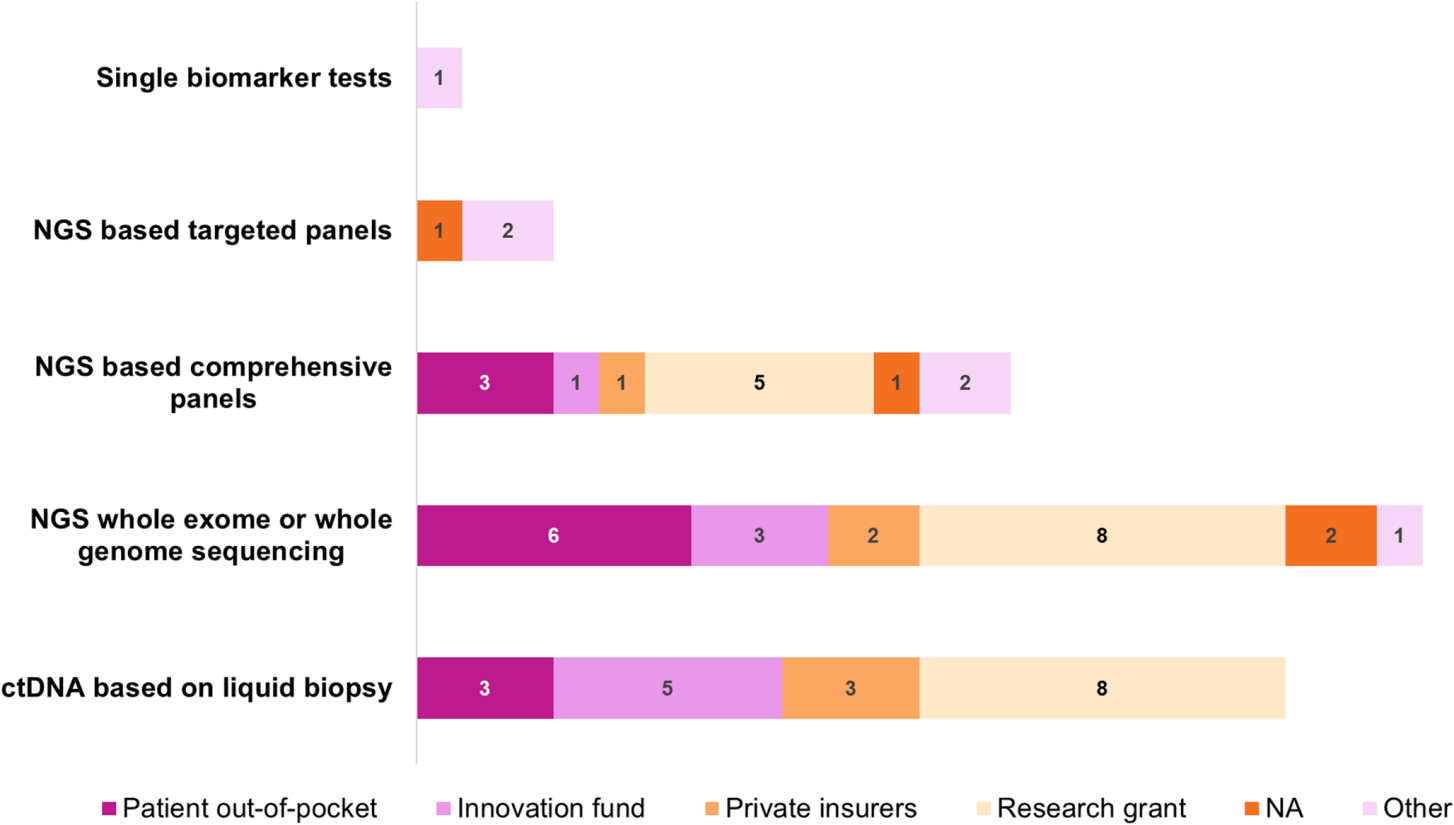
Overview of alternative funding sources for extended molecular diagnostics. This figure depicts the alternative funding sources indicated by survey respondents in the case of no reimbursement. Multiple answers were possible. In case of multiple responders per country, responses were consolidated by counting every funding source mentioned only once per country. Free-text answers for other sources of funding include no funding available, single hospital decision, funded by pharmaceutical companies, national contract with NGS provider, otherwise private funding or through clinical trials. Abbreviations: NGS = next-generation sequencing, ctDNA = circulating tumour DNA

Survey results indicated that targeted panels (covering up to a few dozen genes) were widely reimbursed and usually implemented in routine clinical practice (Fig. 3, panel A). In contrast, comprehensive panels (Fig. 3, panel B) were reimbursed in fewer countries and more frequently used with limitations or only in research settings. Whole genome sequencing was reimbursed in less than half of the countries, mostly not in routine use e.g. research (Fig. 3, panel C). The analysis of ctDNA, where we did not differentiate between targeted or comprehensive genomic profiling, showed mixed implementation status (Fig. 2, panel D). Overall, reimbursement and status of different molecular diagnostics varied considerably. Of note, these results reflect the situation at the time of the survey and potentially are representative for the institution of the respondent rather than for the whole country. Further, extended molecular diagnostics might be used and reimbursed only in case of specific tumour types, upon individual approval, or upon decision of an individual hospital. There was also a considerable diversity in funding sources in the case of no (initial) reimbursement through the national healthcare system or an insurer, including patient out-of-pocket payments, innovation funds, research grants, and private insurers (Fig. 4). Finally, specific health technology assessment processes for diagnostics existed in 10 out of 20 countries, but in 3 of these, no evaluations had been conducted so far (Supplementary Fig. 3).

In implementation projects identified in the literature, funding often came from healthcare insurance systems (Cox et al. 2022; Mukai and Ueno 2021; Taskén et al. 2022; Thompson et al. 2019; Toth et al. 2024; Van Valckenborgh et al. 2018). Other reported funding came from research grants (Bando 2017; Dressler et al. 2019), philanthropy (Rueter et al. 2023), or the budgets of hospitals and cancer research centres (Jalkanen et al. 2024; Mainoli et al. 2024). The exact operationalisation of reimbursement was usually not reported. Genomic tests can be reimbursed through existing codes related to laboratory procedures, often involving code stacking based on the volume of tests performed (Koleva-Kolarova et al. 2022). In inpatient settings, costs can be incorporated in diagnosis-related groups or paid by hospital budgets (Koleva-Kolarova et al. 2022). Inadequate reimbursement, according to literature findings, could be explained by several factors. First, comprehensive gene panels assess hundreds of biomarkers simultaneously and encompass additional activities, such as data interpretation and discussion in molecular tumour boards. Hence, reimbursement via procedural codes for narrowly defined tests can pose challenges (Nadauld et al. 2018). Second, diagnostics in general and extended molecular diagnostics in particular can pose a challenge for health technology assessment that inform reimbursement decisions in many European countries. While test results can link to clear treatment choices, their value may be broader, linked to multiple interventions, guiding trial enrolment, quality of life and improved prognosis (Tarride et al. 2022).

In the interviews, experts addressed funding and reimbursement as a barrier, including disparities in access to reimbursement schemes due to the federalised healthcare system, proximity to university hospitals with higher hospital budgets for molecular analysis, and limited national healthcare budgets. A reimbursement model with the support of pharmaceutical companies and other private sector actors to address limited budgets was suggested. Beyond the routine implementation of molecular diagnostics, one expert mentioned the need for funding to build up infrastructure that resembles clinical trials in terms of collecting follow-up data, and real-world evidence. While out of the scope of this analysis, several experts noted that access to targeted treatments was an important barrier, impacting the clinical value of the molecular analysis if the patient cannot access the treatment. Another recurring theme related to financial infrastructure was the disconnection between the reimbursement of testing and treatment. For example, one expert stated:

Because doctors say that if there is no treatment, why I should pay for quite a lot of money for the testing if I know that anyway there is not treatment available free of charge.

#### Organisational infrastructure

Centralisation of NGS testing, with cancer centres sending samples to a central lab, receiving standardised reports, has been pointed out in the literature as important for implementation due to cost concerns, location of laboratory facilities and the need for skilled personnel and bioinformatics (Hynes et al. 2017). In national and regional infrastructure implementation projects, NGS testing is centralised to either a national centre (Bando 2017; Mainoli et al. 2024) or to a limited number of testing centres (Mukai and Ueno 2021; Rueter et al. 2023), often at university hospitals (Jalkanen et al. 2024; Taskén et al. 2022; Toth et al. 2024). Molecular tumour boards were mentioned in all but one of the identified reports on implementation infrastructure, indicating how central these platforms are. Compared to a traditional tumour board, a molecular tumour board can additionally include biostatisticians, bioinformaticians, molecular biologists, geneticists, researchers and other clinicians (Stoeklé et al. 2018). Tasks include the review of results from extended molecular diagnostics, the matching of patients to biomarker-defined trials, observing the molecular landscape of tumours and notification of new targeted therapies becoming available (Green et al. 2021). They also serve as a forum to share and build expertise (Jain et al. 2021). Administrative support has been implemented in some PCM programs to coordinate trial matching and treatment authorization (Levit et al. 2019; Thompson et al. 2019). The need for a coordinator can be seen as an indicator of the field’s high complexity and interdisciplinarity. Published reviews further highlight the need for PCM implementation strategies that include the perspectives of stakeholders from different sectors (Horgan et al. 2022; Masucci et al. 2024).

In our interviews, centralisation was a recurring theme with slightly varying opinions depending on country size. Experts from countries with smaller population sizes described centralised laboratories in capital cities and considered this sufficient. Experts from larger countries described less centralisation due to regional healthcare organisations. Nevertheless, reducing costs with higher testing volumes, accumulating expertise, and ensuring quality standards, were suggested as drivers for future centralisation. Some experts expressed that referral of patients to centralised analysis is a viable solution, others noted that this might hinder referral, as well as not engaging local physicians. Molecular tumour boards exist in all countries where the experts work, ranging from individual hospital boards to recently established national boards. One expert expressed a need for a national board to coordinate discussion across institutions, while another expressed desire to exchange with other countries. Hence, molecular tumour boards might provide platforms for exchange between institutions within and across countries. Establishing national strategic frameworks with contributions from all stakeholders was mentioned by three experts. It was mentioned that such frameworks can justify funding allocations and streamline implementation efforts. For example, one expert stated:

So it’s really, really important that you have all stakeholders on board, right? To have this collaboration and then such a framework and strategies and action plans in place. And I think this is the most important thing for establishing […] next generation sequencing and personalized medicine related to cancer patients.

#### Competency infrastructure

According to literature, healthcare professionals generally have positive attitudes towards PCM, yet express concerns about insufficient knowledge (Vetsch et al. 2019). Clinicians report less confidence in interpreting and communicating results from extended molecular diagnostics compared to single-gene tests (Blazer et al. 2015). They further report challenges in communication with patients due to unrealistic expectations, the need to communicate uncertainty and assess patients’ knowledge and preferences (Hamilton et al. 2021).

These aspects were also featured in our expert interviews. One interviewed oncologist emphasised that physicians need training to know when to refer patients to molecular tumour boards. Two other highlighted challenges in communication with patients, saying that while having well informed patients that understand treatment possibilities and recommendations is positive, it can be difficult when patients know treatments are inaccessible due to financial constraints. Another expert described that communicating results is challenging due to numerous genomic findings without clinical significance. Solutions mentioned included patient-targeted education through podcasts and lectures, including patient advocates.

The need for highly skilled personnel in both the laboratory and analytical aspects of the diagnostic pathway was emphasized by several experts. Building on existing knowledge from genetic testing was helpful for laboratory protocols, sequencing instruments, and genomic data interpretation. The analytical part requires constant knowledge updates based on evolving trial data and publications. As an example, for the need for highly skilled staff and specific training, one expert stated:

… certainly there are a lot of bioinformaticians and molecular biologists who know a lot about that thing, but they don’t know the healthcare system at all. So, if they can learn more healthcare, then we have a lot more people.

Finally, centralised institutions with good opportunities for continuous learning are important to create an attractive working environment that can compete for highly skilled people. The importance of forming multi-disciplinary teams that collaborate effectively was particularly emphasised.

#### Data infrastructure

Literature suggests data infrastructure to include biobanks for the storage of biopsies, the platforms generating genomic data, a storage base for the genomic data, and a platform producing clinically relevant information from the genomic data through bioinformatic processes (Stoeklé et al. 2018). As more patients undergo comprehensive molecular diagnostics, the large amounts of data will require cloud-based computing infrastructure with consideration for how data can be structured, used, and shared. New models for data sharing and computing capacity are needed for research use (Hinkson et al. 2017). Data must be integrated into reporting systems in a structured, interoperable way using standardised terminology (Chua et al. 2021; Maggi et al. 2019). The special characteristics of genomic data require high levels of protection to guarantee confidentiality, and the validity of patient informed consent for their data to be used for further research (Maggi et al. 2019). As oncology becomes more complex, artificial intelligence provides opportunities to analyse actionable variants (Chua et al. 2021). However, AI implementation places additional requirements on infrastructure, such as frameworks clarifying regulatory aspects, legal liability, and safety standards, continuous validation to reduce bias and additional training for healthcare personnel (Chua et al. 2021).

In practice, current reporting systems are not as structured as needed. Experts described the lack of standardised systems for data structure, the need for a standard-of-care and research testing infrastructure to coexist, and the need for a national solution for storing and sharing genomic data. For example, one expert stated:

So, I think that research is one of the big challenges that we have. For now, everything works well. But again, in five years from now, we will want to transfer our large panel of 400 genes, maybe do whole exome sequencing, and that will involve more expertise, more informatics and support, and more data storage support. And again, the amount of data generated needs to be stored in an appropriate way and needs to be harmonized.

Regarding data sharing between institutions, experts described two challenges: communication requires manual work with information sent via paper, increasing error possibilities; and research data exchange facing bandwidth limitations and legal barriers, especially across borders. One of the experts highlighted the collaboration on clinical trials in different European countries, with improved analysis capabilities including AI tools.

#### Legal infrastructure

While legal infrastructure appeared relatively seldom in our literature search, we identified two exceptions, including the need for a legal framework for innovations such as the development of new actionable or prognostic biomarkers (Mestre-Ferrandiz et al. 2024), informed consent, and differentiating between large panels that could highlight germinal mutations, and narrow diagnostics for treatment definition (Fasola et al. 2023).

Two experts mentioned the link between data and legal infrastructure in our interviews. Data protection laws can hinder research collaborations around genomic data, while infrastructure allowing safe, restricted data access could be improved. A distinction can be made between using patient data for individual benefit versus research benefiting future patients, for example, determining whether previous patients have opted out of their data being used for future research purposes. For example, one expert stated:

… that changes a little bit the way you do diagnostics and also why. […] That you have a broader analysis not because you see a clinical use just to you, but because it’s clinical use for those who come after you that you have more data, […]. But so that’s more of an ethical legal medical aspect.

Legal infrastructure appears interconnected with data infrastructure and requires clarification to facilitate developments such as artificial intelligence and the use of genomic data.

### Summary of results and recommendations on necessary infrastructure

**Fig. 5:**
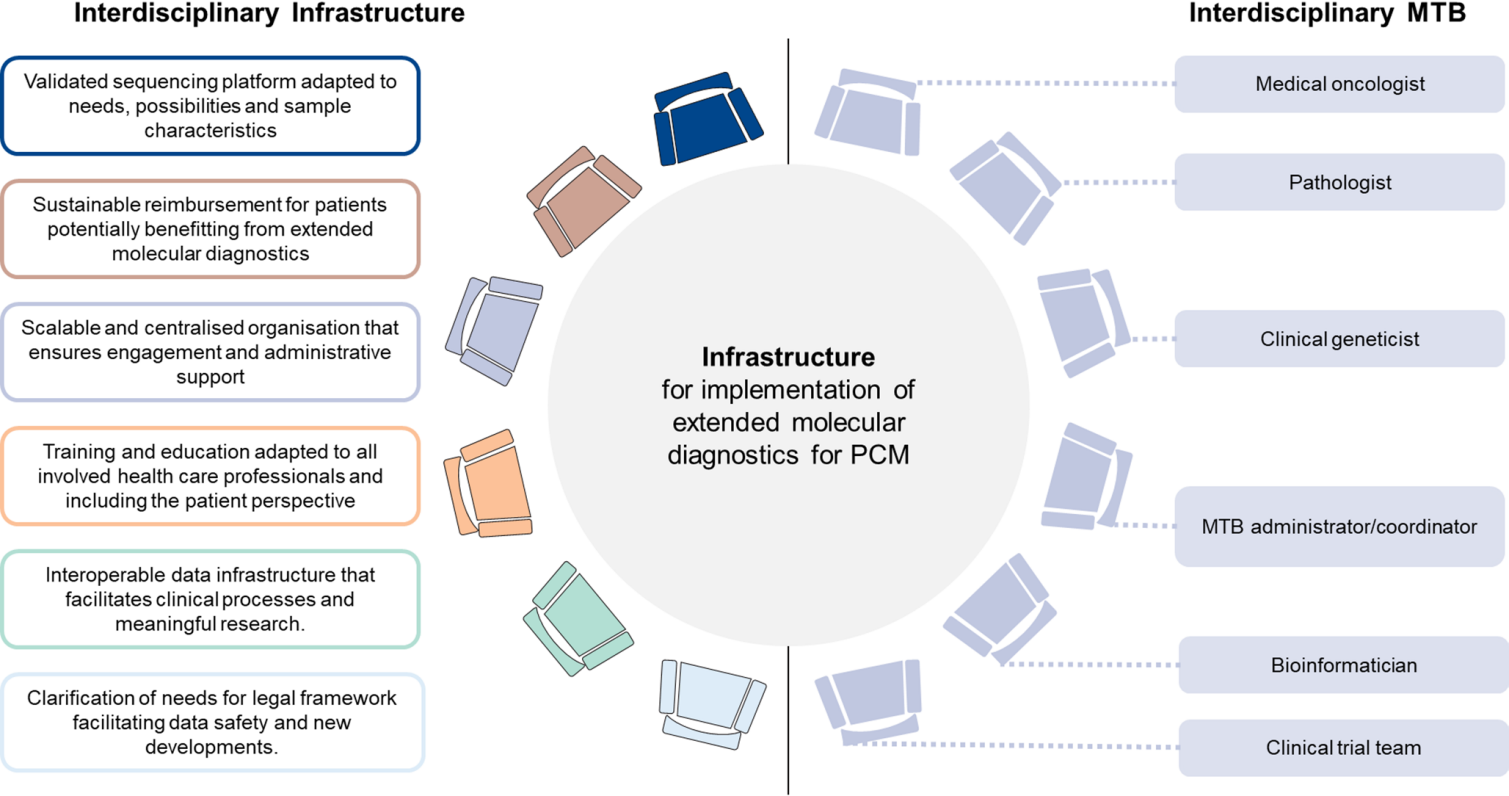
Summary of findings on the necessary infrastructure for the implementation of extended molecular diagnostics for PCM. This figure summarizes the key take-aways based on the findings from the respective categories of infrastructure on the left side. On the right side, the professions typically included in an MTB are represented, drawing a parallel between the multi-disciplinarity collaboration needed in an MTB and across the diagnostic pathway for extended molecular diagnostics. Abbreviations: PCM = precision cancer medicine, MTB = molecular tumour board

Based on the findings per infrastructure categories, recommendations for infrastructure needs per category for the implementation of extended molecular diagnostics for PCM can be derived (Fig. 5). These recommendations highlight the need for multi-disciplinary collaboration across the diagnostic pathway; as such, we draw a parallel to the multi-disciplinary composition in a typical molecular tumour board (Westphalen et al. 2025) (Fig. 5).

## Discussion

Using a mixed-methods approach, we identified six categories of infrastructure required for implementation of PCM: physical, financial, organizational, competency, data and legal infrastructure. While some of our findings reflect the circumstances in individual countries, for instance the size of the population, reimbursement situation and access to treatments, many learnings from infrastructure implementation initiatives in different countries appear generally valid.

Findings from the literature and the expert interviews were generally consistent. The aspect of data infrastructure was more prominent in the expert interviews than what the findings from the literature search suggested. It appears that in practice, evidence collection could be improved by more adequate data infrastructure and means to sharing genomic data, calling for further research. This could facilitate more crucial evidence on the impact and clinical benefit of extended molecular diagnostics in clinical practice (Horgan et al. 2022) and enable a better understanding of which patients benefit the most from targeted treatment approaches. With regards to financial infrastructure, we did not see clear patterns on routine reimbursement of extended molecular diagnostics and characteristics of healthcare systems. However, reimbursement of more comprehensive diagnostics was somewhat more common in western and northern Europe, potentially due to higher healthcare budgets overall. Historically, the costs of genomic sequencing have been falling drastically (van Nimwegen et al. 2016), and could make cost concerns less relevant in the future. Nevertheless, it is obvious that extended molecular diagnostics do not only require the physical infrastructure of laboratories, and sequencing platform, but also other costly infrastructure such as education and training arenas for involved healthcare professionals, as well as structured data systems for sharing and storing of genomic data. In this regard, it should be highlighted that the need for highly skilled staff appeared also a bit more prominent in the interviews than in the literature findings. This raises the question of whether there is a hierarchy of barriers, or an order in which the different categories of infrastructure should be established.

Our results supplement other frameworks for the implementation of PCM. One such framework is the Implementation Framework for PCM Clinical Trials (Carswell et al. 2024) which highlights the role that clinical trials can have in preparing a health system for the large-scale adoption of PCM. Several of the categories mentioned in the frameworks section on preparing a PCM clinical trial are reflected in in our study, i.e., digital ecosystem, financing and reimbursement, and governance and leadership.

### Strengths and limitations of this study

One of our study’s strengths is the reliance on diagnostics as the core prerequisite for PCM. The mixed-methods approach allowed the inclusion of diverse perspectives and data sources. By interviewing experts from countries with different population sizes and different degrees of implementation, we provided an overview of the status and challenges of implementing extended molecular diagnostics in European countries.

Nevertheless, our study had some limitations. Our literature review was structured but not systematic, meaning that we potentially missed relevant publications and that we did not assess the study quality of the included papers. We supplemented our structured review with literature suggested by experts to ensure we included relevant publications. Most of our study informants, including experts suggesting supplementary literature, most interviewees, and the researchers analysing the data, were from the same project consortium, representing large university hospitals and cancer centres, presumably among the most advanced institutions in their countries regarding PCM implementation. While their expertise provided highly relevant input for our study, future research should include additional stakeholders to mitigate this potential bias. For example, experts from smaller institutions might have provided different insights on the topic of centralization, while experts from a payer organization or a manufacturer for laboratory equipment and consumables necessary for molecular diagnostics could have provided more differing views.

Not all European countries were represented in our survey, and contradicting answers sometimes came from within the same country, possibly due to regional differences or survey misunderstandings. Defining reimbursement status across whole countries proved difficult. Our results showcase the varied reimbursement landscape and difficulties in describing the current status, adding to previous studies reporting on accessibility rather than reimbursement (Bayle et al. 2023).

Finally, expanding our methodology to include a systematic literature review, additional survey respondents and interviewees might have enabled us to derive more detailed policy recommendations. However, as our study aimed to explore infrastructure needs for extended molecular diagnostics, we believe that the identified core elements can guide future research about infrastructure needs described in this study.

### Outlook

Several European countries have advanced in PCM implementation as evidenced by established molecular tumour boards and wide accessibility of targeted NGS panels. Infrastructure initiatives and action plans have been important. Since our structured literature review, there have been new publications detailing efforts in countries like France to incorporate genomic sequencing into healthcare systems, particularly for rare diseases and cancers (PFMG contributors 2025), highlighting the rapid advancements in this area. Nevertheless, access to extended molecular diagnostics remains limited and varies across Europe. Challenges such as sustainable reimbursement models, stakeholder engagement and awareness, and interoperable data systems for efficient clinical processes and research persist.

## Conclusion

Implementation of extended molecular diagnostics for PCM requires physical, financial, organisational, competency, data, and legal infrastructure. Our findings emphasise that interdisciplinary collaboration is essential throughout the diagnostic pathway. Significant work remains to provide extended molecular diagnostics to all European patients for whom precision diagnostics are recommended.

## Supplementary Information

Below is the link to the electronic supplementary material.

Supplementary Material 1

Supplementary Material 2

Supplementary Material 3

Supplementary Material 4

## Data Availability

The expert interviews and survey were conducted based on the agreement with participants that data will only be shared in aggregated and anonymous form as reported in this article and in the Supplemental Material.
