# Supplementary material for: Precision cancer medicine in Europe: a mixed-methods study on infrastructure for extended molecular diagnostics
Source: J Cancer Res Clin Oncol. 2026 Apr 2;152(4):80. doi: 10.1007/s00432-026-06468-y (PMC13046898; doi:10.1007/s00432-026-06468-y)
Supplement: Supplementary file 1 — Supplementary Material 1 [file 432_2026_6468_MOESM1_ESM.pdf]

Introduction and survey goals:

With this survey, we want to understand how different types of diagnostics for precision cancer medicine are reimbursed in different countries. We also want to know if these diagnostics undergo a health technology assessment process like pharmaceuticals. Finally, we want to understand what respondents think to be the main barriers beyond potential reimbursement barriers of precision cancer medicine.

This survey is a part of the EU-project PCM4EU, Personalized Cancer Medicine for all EU citizens (http://www.pcm4eu.eu/), funded through EU4Health. The responses will be analyzed by researchers at Oslo University Hospital, and the results will be used in a publication on the implementation of precision cancer medicine.

Thank you for participating!

About you

In this section, we will ask you some questions about yourself and your role in precision cancer medicine

1. In which country do you work?

2. What institution do you work at?

3. What is your role?

- ○ Oncologist
- ○ Pathologist
- ○ Molecular biologist
- ○ Lab engineer
- ○ Clinical coordinating role
- ○ Health economist
- ○ Patient advocate/representative
- ○ Academia/researcher
- ○ Patient advocate/representative
- ○ Other (please specify)

4. How many years of experience in diagnostics of precision cancer medicine do you have?

- ○ < 1 year
- ○ 1-3 years
- ○ 3 – 5 years
- ○ > 5 years

Access to single biomarker tests

The following questions will deal with access to and reimbursement of single biomarker tests (for specific tumor types according to guidelines)

5. How are single biomarker tests used in oncology in your country/region/hospital?

- ◯ Not in routine use
- ◯ In routine oncological practice
- ◯ Only in research
- ◯ Don’t know
- ◯ Other (please specify)

Reimbursement of single biomarker tests

6. Are single biomarker tests reimbursed in your country/region/hospital?

- ◯ Yes, they are reimbursed by the national healthcare system or an insurer
- ◯ No, they are not reimbursed

Information on reimbursement of single biomarker tests

7. Please provide the reimbursement code or name of the reimbursement catalogue for single biomarker tests:

Other financing of single biomarker tests

8. How are single biomarker tests financed in your country/region/hospital? (please choose all the answers that apply)

- ☐ Patient out-of-pocket
- ☐ Innovation fund
- ☐ Private insurers
- ☐ Research grant
- ☐ Other (please specify)

Access to NGS based targeted panel

The following questions will deal with access to and reimbursement of next generation sequencing (NGS) based targeted panels (typically up to a few dozen genes) (for specific tumor types according to guidelines)

9. How are NGS based targeted panels used in oncology in your country/region/hospital?

- ◯ Not in routine use
- ◯ Only in research
- ◯ In routine oncological practice
- ◯ Don’t know
- ◯ Other (please specify)

Reimbursement of NGS based targeted panel

10. Are NGS targeted panels reimbursed in your country/region/hospital?

- ◯ Yes, they are reimbursed by the national healthcare system or an insurer
- ◯ No, they are not reimbursed

Information on reimbursement of NGS based targeted panels

11. Please provide the reimbursement code or name of the reimbursement catalogue for NGS based targeted panels

Other financing of NGS based targeted panels

12. How are NGS based targeted panels financed in your country/region/hospital? (please choose all the answers that apply)

- ☐ Patient out-of-pocket
- ☐ Innovation fund
- ☐ Private insurers
- ☐ Research grant
- ☐ Other (please specify)

Access to NGS based comprehensive panels

The following questions will deal with access to and reimbursement of NGS based comprehensive panels (several hundred genes) (for specific tumor types according to guidelines)

13. How are NGS based comprehensive panels used in oncology in your country/region/hospital?

- ○ Not in routine use
- ○ In routine oncological practice
- ○ Only in research
- ○ Don’t know
- ○ Other (please specify)

Reimbursement of NGS based comprehensive panels

14. Are NGS comprehensive panels reimbursed in your country/region/hospital?

- ◯ Yes, they are reimbursed by the national healthcare system or an insurer
- ◯ No, they are not reimbursed

Information on reimbursement of NGS based comprehensive panels

15. Please provide the reimbursement code or name of the reimbursement catalogue for NGS based comprehensive panels

Other financing of NGS based comprehensive panels

16. How are NGS based comprehensive panels financed in your country/region/hospital? (please choose all the answers that apply)

- [ ] Patient out-of-pocket
- [ ] Innovation fund
- [ ] Private insurers
- [ ] Research grant
- [ ] Other (please specify)

Access to NGS whole exome or whole genome sequencing

The following questions will deal with access to and reimbursement of NGS whole exome or whole genome sequencing (for specific tumor types according to guidelines)

17. How is NGS whole exome or whole genome sequencing used in oncology in your country/region/hospital?

- ◯ Not in routine use
- ◯ In routine oncological practice
- ◯ Only in research
- ◯ Don’t know
- ◯ Other (please specify)

Reimbursement of NGS whole exome or whole genome sequencing

18. IS NGS whole exome or whole genome sequencing reimbursed in your country/region/hospital?

- ◯ Yes, it is reimbursed by the national healthcare system or an insurer
- ◯ No, it is not reimbursed

Information on reimbursement of NGS whole exome or whole genome sequencing

19. Please provide the reimbursement code or name of the reimbursement catalogue for NGS whole exome or whole genome sequencing:

Other financing of NGS whole exome or whole genome sequencing

20. How is NGS whole exome or whole genome sequencing financed in your country/region/hospital? (please choose all the answers that apply)

- ☐ Patient out-of-pocket
- ☐ Innovation fund
- ☐ Private insurers
- ☐ Research grant
- ☐ Other (please specify)

Access to ctDNA based on liquid biopsy

The following questions will deal with access to and reimbursement of circulating tumor DNA (ctDNA) based on liquid biopsy (for specific tumor types according to guidelines)

21. How is circulating tumor DNA (ctDNA) based on liquid biopsy used in oncology in your country/region/hospital?

- Not in routine use
- In routine oncological practice
- Only in research
- Don’t know
- Other (please specify)

Reimbursement of ctDNA based on liquid biopsy

22. Is ctDNA based on liquid biopsy reimbursed in your country/region/hospital?

- ◯ Yes, it is reimbursed by the national healthcare system or an insurer
- ◯ No, it is not reimbursed

Information on reimbursement of ctDNA based on liquid biopsy

23. Please provide the reimbursement code or name of the reimbursement catalogue for ctDNA based on liquid biopsy

Other financing of ctDNA based on liquid biopsy

24. How is ctDNA based on liquid biopsy financed in your country/region/hospital? (please choose all the answers that apply)

- [ ] Patient out-of-pocket
- [ ] Innovation fund
- [ ] Private insurers
- [ ] Research grant
- [ ] Other (please specify)

Health technology assessment (HTA)

Health Technology Assessment (HTA) describes a systematic evaluation of the medical, economic, social, and ethical properties of health technologies. In many European countries, HTA is used to support decision-making for reimbursement and pricing of health technologies.

25. Is there a specific HTA process in place in your country that is applied to diagnostics for precision cancer medicine?

- ◯ Yes
- ◯ No
- ◯ Don’t know

Previous HTA evaluations

26. Have previous HTA evaluations of diagnostics for precision cancer medicine been conducted and published by a national authority in your country?

- ◯ No
- ◯ Yes, but I cannot provide information on this evaluation
- ◯ Yes, and I can provide a link to this report and/or the name of the national authority:

- ◯ Don't know

Implementation of precision cancer medicine in your country

27. Beyond potential barriers resulting from lack of reimbursement, what do you perceive as the most important barriers for the implementation of precision cancer medicine as part of the healthcare system?

Additional comments

28. Do you have any additional comments you would like to add regarding the reimbursement of diagnostics for PCM?
