# Supplementary material for: Precision cancer medicine in Europe: a mixed-methods study on infrastructure for extended molecular diagnostics
Source: J Cancer Res Clin Oncol. 2026 Apr 2;152(4):80. doi: 10.1007/s00432-026-06468-y (PMC13046898; doi:10.1007/s00432-026-06468-y)
Supplement: Supplementary file 2 — Supplementary Material 2 [file 432_2026_6468_MOESM2_ESM.pdf]

Infrastructure needed for implementation of diagnostics for precision cancer medicine as part of the health care system

Interview questions for semi-structured Expert interviews

Short project description

We are conducting a mixed-methods study on the necessary infrastructure for extended molecular diagnostics in precision cancer medicine (PCM). The aim is to get an overview of the challenges and provide recommendations related to the implementation of extended molecular diagnostics for PCM as part of health care systems in Europe. The insights from this study will be summarized in a whitepaper, deliverable 4.3 in work package 4 of the PCM4EU (Personalized Cancer Medicine for all EU citizens) project. As part of this study, we are conducting expert interviews with consortium members of the PCM4EU and ASCERTAIN project and affiliated experts.

Topic 1: current and future use of diagnostics for PCM

- How are diagnostics for precision cancer medicine used in your institution or your country?
- How is a decision on the use of next-generation-sequencing/comprehensive genomic profiling made?
- How do you think the use will change in the next 5-10 years?

Topic 2: Infrastructure

- Which infrastructure do you think is necessary to facilitate implementation? Why?
- Which digital infrastructure is needed to implement PCM as part of the healthcare system? For example, possibilities for data storage, secure communication, and online meeting channels?

Topic 3: Upscaling capacity and the pathway to routine care

- How could precision cancer diagnostics currently established in research settings become a part of routine care?
- How could test capacity in terms of number of patient samples be increased?

Topic 4: Reimbursement and funding

- How do reimbursement and funding processes influence the use of diagnostics for precision cancer medicine?

Topic 5: Organization of PCM

- How should implementation of PCM diagnostics be organized? Why?

Topic 6: Barriers

- What do you perceive as barriers currently impeding the implementation of precision cancer medicine as part of the healthcare system?
- What is the single most important barrier?

Closure: best practice sharing

- How could your institution or your country learn from others in the implementation process?

Is there anything else I should have asked?

Any questions from the interviewee?

Version 1.5 from July 23, 2024
