# Supplementary material for: Precision cancer medicine in Europe: a mixed-methods study on infrastructure for extended molecular diagnostics
Source: J Cancer Res Clin Oncol. 2026 Apr 2;152(4):80. doi: 10.1007/s00432-026-06468-y (PMC13046898; doi:10.1007/s00432-026-06468-y)
Supplement: Supplementary file 3 — Supplementary Material 3 [file 432_2026_6468_MOESM3_ESM.pdf]

# Search query for necessary infrastructure for implementation of extended molecular diagnostics into healthcare systems

**Medline via PubMed**

1
"High-Throughput Nucleotide Sequencing"[Mesh:NoExp] OR “Molecular Diagnostic Techniques"[Mesh:NoExp] OR Next Generation Sequencing[Title]

2
"Precision Medicine"[Mesh:NoExp] OR ((precision[Title] OR personalized[Title] OR personalised[Title] OR individualized[Title] OR individualised[Title] OR stratified[Title] OR theranostic*[Title] OR theragnostic*[Title] OR P4[Title]) AND (medicine[Title] OR nanomedicine[Title]))

3
Neoplasms[Mesh:NoExp] OR Medical Oncology[Mesh] OR cancer*[Title] OR oncol*[Title]

4
"precision cancer"[Title] OR "precision oncology"[Title]

5
"Delivery of Health Care"[Mesh:NoExp] OR "Health Services"[Mesh:NoExp] OR Infrastruc*[Title] OR implement*[Title] OR health care[Title] OR healthcare[Title] OR health system[Title] OR health systems[Title] OR health organization[Title] OR health organizations[Title] OR health organisation[Title] OR health organisations[Title] OR health service[Title] OR health services[Title] OR "Quality Improvement"[Mesh] OR "Guideline Adherence"[Mesh] OR "Quality Improvement"[Title]

The search strings below are combined as follows: ((1 OR 2) AND (3) OR 4) AND (5)

(("High-Throughput Nucleotide Sequencing"[Mesh:NoExp] OR “Molecular Diagnostic Techniques"[Mesh:NoExp] OR Next Generation Sequencing[Title] OR "Precision Medicine"[Mesh] OR ((precision[Title] OR personalized[Title] OR personalised[Title] OR individualized[Title] OR individualised[Title] OR stratified[Title] OR theranostic*[Title] OR theragnostic*[Title] OR P4[Title]) AND (medicine[Title] OR nanomedicine[Title]))) AND (Neoplasms[Mesh:NoExp] OR Medical Oncology[Mesh] OR cancer*[Title] OR oncol*[Title]) OR "precision cancer"[Title] OR "precision oncology"[Title]) AND ("Delivery of Health Care"[Mesh:NoExp] OR "Health Services"[Mesh:NoExp] OR Infrastruc*[Title] OR implement*[Title] OR health care[Title] OR healthcare[Title] OR health system[Title] OR health systems[Title] OR health organization[Title] OR health organizations[Title] OR health organisation[Title] OR health organisations[Title] OR health service[Title] OR health services[Title] OR "Quality Improvement"[Mesh] OR "Guideline Adherence"[Mesh] OR "Quality Improvement"[Title]) AND "last 10 years"[PDat]

Retrieves 305 results on July 16, 2024

**Embase Classic + Embase via Ovid:**

1
high throughput sequencing/ OR molecular diagnostics/ OR next generation sequencing.ti.

2
exp personalized medicine/ OR ((precision.ti. OR personalized.ti. OR personalized.ti. OR individualized.ti. OR individualized.ti. OR stratified.ti. OR theranostic*.ti. OR theragnostic*.ti. OR P4.ti.) AND (medicine.ti. OR nanomedicine.ti.))

3
neoplasm/ OR oncology/ OR cancer*.ti. OR oncol*.ti.

4
'precision cancer'.ti. OR 'precision oncology'.ti.

5
health care delivery/ OR exp health care facilities and services/ OR Infrastruc*.ti. OR implement*.ti. OR health care.ti. OR healthcare.ti. OR health system.ti. OR health systems.ti. OR health organization.ti. OR health organizations.ti. OR health organization.ti. OR health organisations.ti. OR health service.ti. OR health services.ti. OR total quality management/ OR protocol compliance/ OR Quality Improvement.ti.

The search strings below are combined as follows: ((1 OR 2) AND (3) OR 4) AND (5)

((high throughput sequencing/ OR molecular diagnostics/ OR next generation sequencing.ti. OR exp personalized medicine/ OR ((precision.ti. OR personalized.ti. OR personalized.ti. OR individualized.ti. OR individualized.ti. OR stratified.ti. OR theranostic*.ti. OR theragnostic*.ti. OR P4.ti.) AND (medicine.ti. OR nanomedicine.ti.))) AND (neoplasm/ OR oncology/ OR cancer*.ti. OR oncol*.ti.) OR 'precision cancer'.ti. OR 'precision oncology'.ti.) AND (health care delivery/ OR exp health care facilities and services/ OR Infrastruc*.ti. OR implement*.ti. OR health care.ti. OR healthcare.ti. OR health system.ti. OR health systems.ti. OR health organization.ti. OR health organizations.ti. OR health organization.ti. OR health organisations.ti. OR health service.ti. OR health services.ti. OR total quality management/ OR protocol compliance/ OR Quality Improvement.ti.)

Limit to to yr="2014 -Current"

Retrieves 556 results on July 16, 2014
