# Supplementary material for: Precision cancer medicine in Europe: a mixed-methods study on infrastructure for extended molecular diagnostics
Source: J Cancer Res Clin Oncol. 2026 Apr 2;152(4):80. doi: 10.1007/s00432-026-06468-y (PMC13046898; doi:10.1007/s00432-026-06468-y)
Supplement: Supplementary file 4 — Supplementary Material 4 [file 432_2026_6468_MOESM4_ESM.pdf]

Supplementary Tables & Figures to the article

Precision cancer medicine in Europe: a mixed-methods study on infrastructure for extended molecular diagnostics

Journal of Cancer Research and Clinical Oncology
Pia S. Henkel, Kine Pedersen, Kjetil Taskén, Ebba Hallersjö Hult, Hans Gelderblom, G. Live Fagereng, Helga B. Landsverk, Eline Aas
Corresponding author: Pia S. Henkel, p.s.henkel@medisin.uio.no, Institute of Health and Society, University of Oslo, Oslo, Norway

Supplementary Table 1: **Countries represented by number of survey respondents.** This table depicts the self-reported country of work of the survey participants. The survey was distributed through the PCM4EU network and ASCERTAIN network (see www.pcm4eu.eu and https://www.access2meds.eu/ for details on participating partners) and open between June 10 and July 29, 2024.

Country	# responders	% responders
Belgium	1	2 %
Croatia	2	4 %
Czech Republic	1	2 %
Denmark	3	6 %
Estonia	3	6 %
Finland	1	2 %
France	1	2 %
Germany	5	11 %
Hungary	2	4 %
Italy	2	4 %
Ireland	2	4 %
Lithuania	3	6 %
Netherlands	2	4 %
Norway	4	9 %
Poland	4	9 %
Portugal	2	4 %
Slovakia	1	2 %
Spain	1	2 %
Sweden	3	6 %
United Kingdom	1	2 %
Not indicated and excluded from further analysis	3	6 %
**Total**	**47**	**100 %**

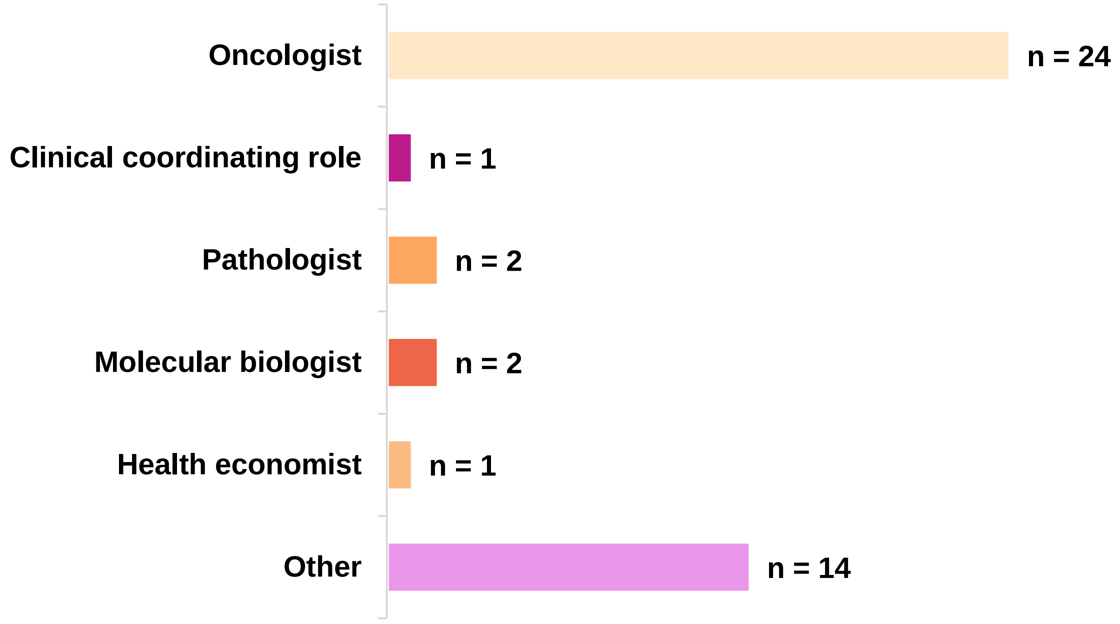

Supplementary Figure 1: Number of survey respondents per profession. This figure depicts the self-reported professions of the survey participants. Other professions indicated include geneticist, project coordinating roles, research, and director roles.

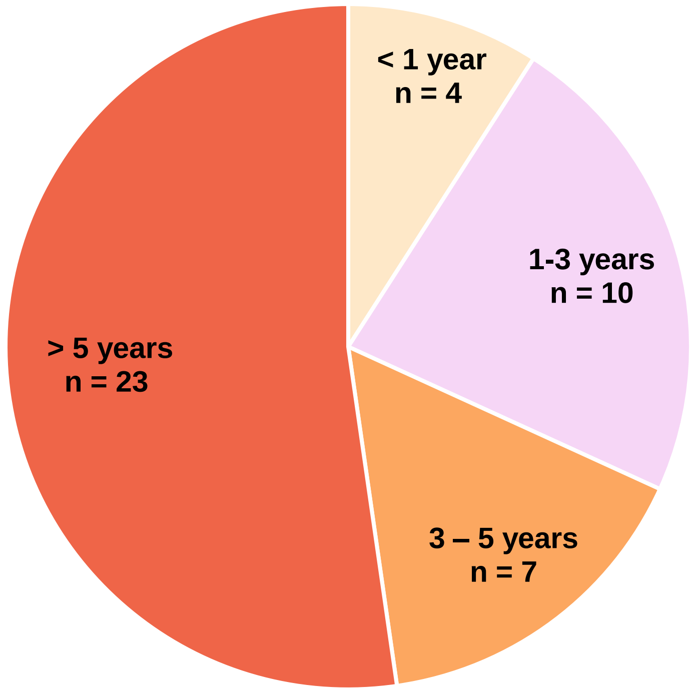

Supplementary Figure 2: Survey respondents experience with precision cancer medicine. This figure depicts the self-reported number of years of experience with precision cancer medicine of the survey participants.

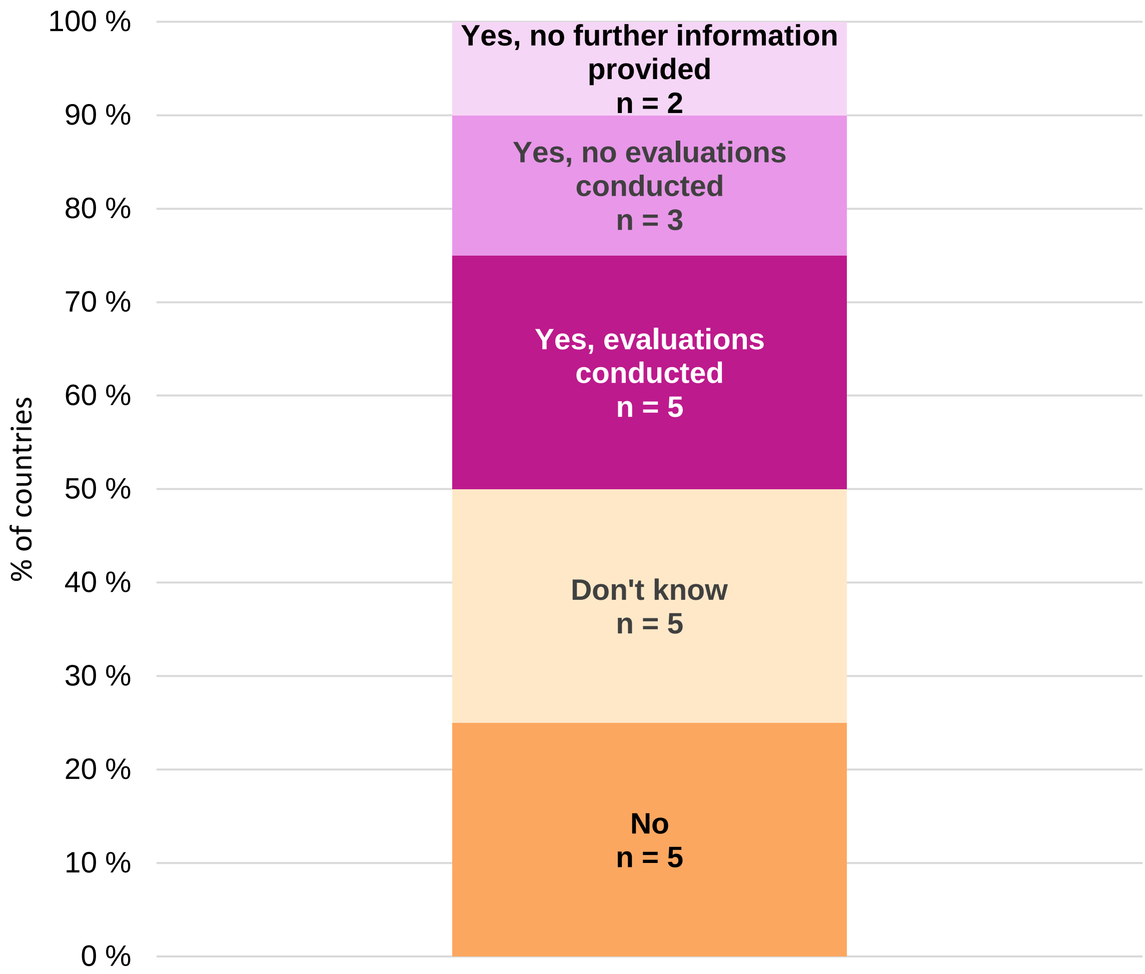

Supplementary Figure 3. Overview of the existence of an HTA process for diagnostics in 20 European countries. This figure depicts the number of countries for which survey respondents answered that a health technology assessment (HTA) process for diagnostics is in place. Respondents were further asked if HTA evaluations have previously been conducted for diagnostics for precision cancer medicine. In case of multiple responders per country, responses were consolidated, reporting a “Yes” if at least one respondent answered with “Yes”.

Supplementary table 2: Open-ended survey answers on barriers for implementation.
This table lists the answers received to the question “Beyond potential barriers resulting from lack of reimbursement, what do you perceive as the most important barriers for the implementation of precision cancer medicine as part of the healthcare system?”
* If several aspects were mentioned in the same answer, the answer was split in two or more answers

Answers from survey
Lack of agreement to whom comprehensive testing should be offered/reimbused, lack of guidelines
Lack of agreement between different parties, lack of guidelines to whom comprehensive (exome) testing should be applied
* data analysis (when and how collected, harmonised between hospitals within country)
clinical interpretation and PCM education, *
Critical mass of expertise, *
lack of knowledge
clinicians thinking only about the drugs available now, there is no view of the future. No understanding of data storage etc..
Lack of high level evidence
knowledge about the real benefit of these tests is largely lacking
* Physical infrastructure, *
availabilty of genetic testing and *
Lack of cost effective diagnostic infrastructure
heterogeneous access in different hospitals/regions
capability of hospital's IT system in integrating all the results of different biomarkers
money and infrastructure dedicated to diagnostics
* no national MTB
Federated structure of health care system
lack of nation-wide standardized workflow for precision cancer diagnostics
decentralized labs
Our contry has a regionalized healthcare system which means that each region decides independently on implementation of precision diagnostics/medicine.
A lack of a national strategy to cover not only costs but all other aspects of clinical implementation of precision cancer medicince.
* appropriately trained personnel
lack of personnel, long turnaround time, insufficient tissue, administrative load
Additional Funding - Workforce and education

Lack of reimbursement and IVDR regulation
The reimbursement of both comprehensive genomic profiling and targeted therapies
* availability of innovative treatment, new clinical trials
low availability of innovative medicines, low number of clinical trials, *
availability of the precision cancer therapy (reimbursement)
Lack of access to therapies
the Danish medicines council - are relevant drugs approved?
the access to treatment
The access to targeted drugs. The trageted drugs are available but it is difficult to access them off-label due to restricted reimbursement of the drugs.

Supplementary table 3: Examples of quotes per category referring to recurring themes in the interviews. This table lists examples of quotes with recurring themes from the expert interviews. The first column lists the recurring theme, the second column lists the infrastructure category that this theme was classified into and the third column contains the exemplary quotes.

Recurring theme	Categorization to an infrastructure category	Quotes
Contextualization of the choice of analysis technology	Physical infrastructure	"And the problem is how we could collect the minimum amount of patient samples. Because they also want to have the reports quite quickly back and then we decided to specifically go for the biggest panel, most expanded panel just to combine different samples."<br><br>"For instance, it's not the same the amount of tissue that you need to perform the panel that we do. We have everything optimized to do it in samples, in real-world samples, meaning the ones that the patient provides in paraffin and the blocks."<br><br>"So, everything is optimized now to do the best that we can for the patients that we have based on the capabilities that we have."
Funding and reimbursement as barriers	Financial infrastructure	"But if we are talking that we should have a bigger number of oncological patients or tumor samples what should be tested then I think that still it's the biggest barrier is the money."<br><br>"The basic framework is the same, meaning that the numbers that have been put out there for reimbursement, equal whether you are living in [name of a city] and [name of another city] or in [name of a third city], but the access to these reimbursement schemes can differ from center to center."<br><br>"They [referring to means to fund tests] come directly from the hospital budget, not like from the genomic center or genome center, which should, in my opinion, be more systematic so that everybody had access, non-dependent on where the patient is living. Otherwise, it's more tendency to the university hospital side. And as you know, [country name] is a very big country, so there are quite a lot of patients who may have even 400 kilometers to the nearest university hospital. So it's not easy."

Disconnect between the reimbursement of testing and treatment	Financial infrastructure	"Because doctors say that if there is no treatment, why I should pay for quite a lot of money for the testing if I know that anyway there is not treatment available free of charge"<br><br>"… the question of how well testing is reimbursed is not transparent or sometimes even not discussed at all. So it's just discussed with respect to the cost effectiveness of the drug."<br><br>"… because people are so keen on having reimbursement for something, but we're not discussing the reimbursement actually for the diagnostic side itself. It's sort of thought that it's an automation. But if they're not having any increase in their resources, it's just not going to happen."
Centralization of testing	Organizational infrastructure	"And the population of [country name] is only [country population] million. So I think that usually if we think about genetic services, they say that that one genetic center per one to two million of population."<br><br>"So, on the one hand, centralization delivers high standards on genomics, the very same level, it's very scalable. And on the other hand, you need to take care of people at the local sites who are treating the patient, who provide consulting to the families and so on and so forth. So it's really, I think, a fine balance and the network needs to account for this."<br><br>"In other countries, it is not centralized. There are more centers. Of course, it depends on the country size. [Country name] is a small country. I think that in those very rare cases, we should not spread so much. We have to make those one or two centers in [country name] not to spread so much, because we will not gather information, we will not accumulate it, and I think it's not the way to go."
Establishing national strategic frameworks	Organizational infrastructure	"So it's really, really important that you have all stakeholders on board, right? To have this collaboration and then such a framework and strategies and action plans in place. And I think this is the most important thing for establishing of all needed for such next generation sequencing and personalized medicine related to cancer patients."

		"And we are speaking about how we should get together and not to work everybody by themselves, not to spread our infrastructure. But to get together and to have this national like strategy how we are working …"<br><br>"And then it's important to have a national strategy, you know, as a foundation pointing, this is already in the white paper said that we need this, and it's already decided that by the government that this is what we will do so that you don't have to reinvent the wheel all the time."
Need for highly skilled staff	Competency infrastructure	"there constantly needs to be human labor work looking at only and interpreting the data so that it's fully matched and up to date as well"<br><br>"And even the surgeon, because they're the ones that basically, if you need fresh tumor tissue, so that's upon diagnostic, very important that the tissue itself is handled appropriately."<br><br>"… certainly there are a lot of bioinformaticians and molecular biologists who know a lot about that thing, but they don't know the healthcare system at all. So, if they can learn more healthcare, then we have a lot more people. So how to train them in health care, you know, in being a health care personnel, that is definitely not on the molecular biology training."
Lack of systems and use of data for research	Data infrastructure	"We have the electronic system where all the clinical data … If you are patient, you can look after all your [unintelligible] and reports and test reports and so on. But in the same time, they are not structured"<br><br>"So it is important that we have also appropriate registries for collecting real world data and that we use also real world data in connection with the current study designed to provide appropriate evidence for further assessment and decision making related to precision cancer medicine and diagnostic tests."<br><br>"… if we will start this testing, a big amount of testing, then we will have to think what to do with this big data. How to safely store them,

		what, how to use them, how to share them maybe with the other institutions and in the country, the data sharing issue and the privacy issues are very important.”<br><br>“So I think that research is one of the big challenges that we have. For now, everything works well. But again, in five years from now, we will want to transfer our, our large panel of 400 genes, maybe do whole exome sequencing, and that will involve more expertise, more informatics and support, and more data storage support. And again, the amount of data generated needs to be stored in an appropriate way and needs to be harmonized.”<br><br>“I think that you need to just acknowledge that there is a need to think both for the specific patient and the collection of data for the future patients. And that's just a different way to work.”
Data protection and using patient data for future research	Legal infrastructure	“I mean, between two countries always has quite some obstacles in terms of the legal framework. So there are multiple national laws beside the EU law which would equally apply in all of these countries but there is a lot of national law. And I think this greatly hinders cross country, collaborative work, starting with barriers surrounding the exchange of data, for example.”<br><br>“And there has been a lot of legal work on what makes a genetic variant anonymous or not. And sort of still ongoing a little bit. Because, of course, with rare diseases, it's always a geneticist to remember the family with a particular genetic variant, you know, so it's not anonymous very easily.”<br><br>“And then, of course, that changes a little bit the way you do diagnostics and also why. So that is also a legal and ethical question, of course. That you have a broader analysis not because you see a clinical use just to you, but because it's clinical use for those who come after you that you have more data, […]. But so that's more of an ethical legal medical aspect.”
